# Developing anti-tobacco messages for Australian Aboriginal and Torres Strait Islander peoples: evidence from a national cross-sectional survey

**DOI:** 10.1186/1471-2458-14-250

**Published:** 2014-03-13

**Authors:** Gillian S Gould, Kerrianne Watt, Leah Stevenson, Andy McEwen, Yvonne Cadet-James, Alan R Clough

**Affiliations:** 1 School of Public Health, Tropical Medicine and Rehabilitation Sciences, James Cook University, PO Box 6811, Cairns, Queensland 4870, Australia; 2 Southern Cross University, Hogbin Drive, Coffs Harbour, NSW 2450, Australia; 3 School of Public Health, Tropical Medicine and Rehabilitation Sciences, James Cook University, Townsville, Queensland 4811, Australia; 4 Health Behaviour Research Centre, Epidemiology and Public Health, University College London, London, UK; 5 School of Indigenous Australian Studies, James Cook University, Townsville, Queensland, Australia

**Keywords:** Aboriginal and Torres Strait Islander peoples, Oceania ancestry group, Tobacco smoking, Anti-tobacco messages, Smoking cessation, Cultural sensitivity, Targeted health promotion messages, evaluation

## Abstract

**Background:**

Smoking rates in Australian Aboriginal and Torres Strait Islander peoples remain high, with limited impact of government measures for many subgroups. The aim of this cross-sectional study was to investigate differences in organisational practice for developing anti-tobacco messages for these target populations.

**Methods:**

Telephone interviews were conducted with 47 organisation representatives using a structured questionnaire based on health communication and health promotion frameworks. Responses were coded into phases of message development, message types (educational, threat, positive or advocacy), target groups, message recommendations, and evaluations undertaken. Cultural sensitivity for message development was divided into surface structure (use of images, language, demographics) and deep structure (use of socio-cultural values). A categorical principal component analysis explored the key dimensions of the findings and their component relationships.

**Results:**

Among organisations interviewed, a community-orientated, bottom-up approach for developing anti-tobacco messages was reported by 47% (n = 24); 55% based message development on a theoretical framework; 87% used a positive benefit appeal; 38% used threat messages. More Aboriginal Medical Services (AMSs) targeted youth (p < 0.005) and advised smokers to quit (p < 0.05) than other types of organisations. AMSs were significantly more likely to report using deep structure in tailoring messages compared with non-government (p < 0.05) and government organisations (p < 0.05). Organisations that were oriented to the general population were more likely to evaluate their programs (p < 0.05). A two-dimensional non-linear principal component analysis extracted components interpreted as “cultural understanding” (bottom-up, community-based approaches, deep structures) and “rigour” (theoretical frameworks, and planned/completed evaluations), and accounted for 53% of the variability in the data.

**Conclusion:**

Message features, associated with successful campaigns in other populations, are starting to be used for Aboriginal and Torres Strait Islander peoples. A model is proposed to facilitate the development of targeted anti-tobacco messages for Aboriginal and Torres Strait Islander peoples. Organisations could consider incorporating both components of cultural understanding-rigour to enable the growth of evidence-based practice.

## Background

In Australia, smoking in the general population has steadily decreased since the introduction of media campaigns, smoke-free legislation and pricing increases [1]. It is currently estimated at 15% [2]. Smoking prevalence in Aboriginal and Torres Strait Islander peoples, whilst also on a downward trend, is 2.6 times that of the general population at 41% [3] and the gap between Aboriginal and Torres Strait Islander daily smokers and the general population has only closed by 2% over the last 10 years [3]. However, daily smoking in Aboriginal and Torres Strait Islander peoples in the 25–34, and 45–54 age groups, and in remote areas, has not declined significantly in the last decade [3].

Factors that contribute to the continued use of tobacco by Aboriginal and Torres Strait Islander peoples include historical use, the effect of colonisation, community norms, and multiple structural and socio-economic inequalities [4]. There are barriers to adequate implementation of tobacco control campaigns in some Aboriginal and Torres Strait Islander communities, especially if remote [4]. Inadequate reach could contribute to lower campaign effectiveness [5]. Moreover there are inequities in health care access and cessation treatments for Aboriginal and Torres Strait Islander peoples [4].

Most mass-media campaign research has been in high income populations, with less emphasis on the special needs of disadvantaged groups such as Indigenous populations [6]. There is limited research into mass media anti-tobacco programs for Aboriginal and Torres Strait Islander peoples with reports of little consistency of approaches [7-9]. In 2011 the National Tobacco Campaign developed the targeted ‘Break the Chain’ TV campaign, to aid Aboriginal and Torres Strait Islander smokers to acknowledge the health impacts from smoking. The campaign aims to reduce by half the prevalence of smoking among Aboriginal and Torres Strait Islander peoples by 2018 [9]. The Council of Australian Governments ‘closing the gap’ strategy is currently devoting significant funding for local anti-tobacco programs for Aboriginal and Torres Strait Islander communities [10].

The Ottawa Charter for Health Promotion recommends that health messages are respectful of the cultural needs of diverse populations [11]. Targeting, a popular strategy for behaviour change, has been used with socially disadvantaged and ethnic minorities, and on the basis of culture. Kreuter and Skinner [12] defined targeting as “the development of a single intervention approach for a defined population subgroup that takes into account characteristics shared by the subgroup’s members.” Although Indigenous peoples have good recall of generic mass media messages these do not necessarily lead to behaviour changes [7]. A recent systematic review indicated that culturally-specific anti-tobacco mass media programs, when used for Indigenous peoples, have been as effective in promoting quitting as generic messages are on the general population, and moreover they are preferred by Indigenous peoples from westernised nations [7]. However, appropriately controlled comparative studies to measure the efficacy of mass media interventions are scarce [7]. There is also limited evidence for the efficacy of cessation interventions specifically targeted to Indigenous peoples [13].

Less attention has been given to the way anti-tobacco messages are developed for Indigenous populations. Health communication research has shown that highly emotive messages are more likely to be effective in anti-tobacco media campaigns [14]. Fear-inducing messages are most often utilised in negative health effects campaigns [5], but positive emotions, such as humour and pride, can be important to prompt attitude and behaviour changes [15]. It is unknown how organisations are currently developing anti-tobacco messages for Aboriginal and Torres Strait Islander communities, what emotion-based messages are being used, or whether message construction has been informed by health communication and behaviour change theories.

With limited published evidence to guide the development of anti-tobacco messages for the Aboriginal and Torres Strait Islander target groups, and the proliferation of many new community based tobacco teams tackling Indigenous smoking, we believed it necessary to gain a clearer picture of current health promotion practices for tobacco in Australia. We aim to (a) understand what processes are being undertaken in making anti-tobacco health messages for Aboriginal and Torres Strait Islander peoples, (b) understand the range of anti-tobacco messages developed, (c) compare differences according to organisational type, and examine the main components accounting for variations in findings. By providing a snapshot of current and recent practices, these findings contribute to important issues for Indigenous tobacco control as outlined in government policies [16,17].

(Terminology: we prefer to use the term Aboriginal and Torres Strait Islander peoples when referring to the Indigenous peoples of Australia. However we also use the term Indigenous when referring to international literature about Indigenous people in general, and about policies (such as Indigenous tobacco control). Where a study has reported only on one people, e.g. Aboriginal, that term is used).

## Methods

### Study design

We surveyed Australian organisations involved in the development of health promotional messages for tobacco control with Aboriginal and Torres Strait Islander peoples. Both quantitative and qualitative data were collected to provide multiple standpoints. Only quantitative findings are reported here, along with a brief qualitative overview of programs. By interviewing one representative from each organisation a degree of intersubjectivity [18] and experiential knowledge was assumed. However we sought functional knowledge to guide future pragmatic considerations for anti-tobacco message development and to open up opportunities for transferability [18].

### Sampling

Potential participating Indigenous tobacco control programs were identified from:

•Australian Indigenous Health Info-net website

•Centre for Excellence in Indigenous Tobacco Control website

•Indigenous Tobacco Control Initiative funding recipients

•Tackling Indigenous Smoking Tobacco Teams

•Contacts recommended by other participants, and those known to investigators

**Figure 1:**
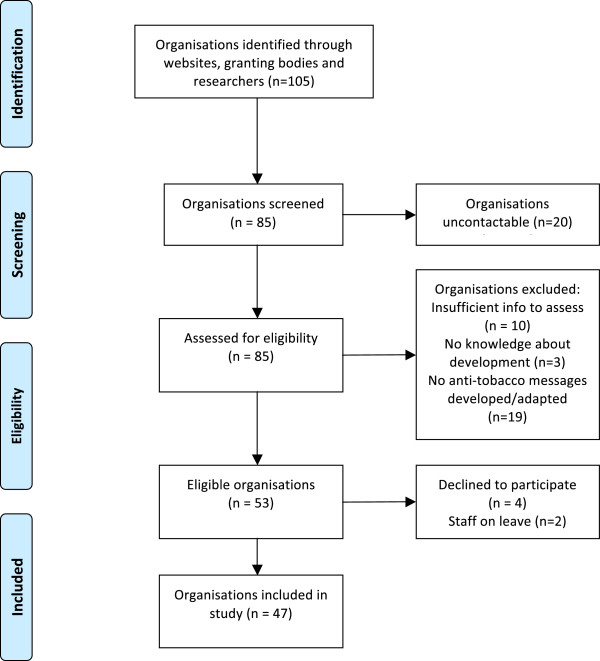
Flow diagram of selection of organisations for the study.

Contact was made by telephone where possible. Information sheet, questionnaire, and consent forms were then emailed to potential participants. Eligibility criteria were: (a) the organisation had to have developed or adapted anti-tobacco messages for Aboriginal and/or Torres Strait Islander peoples; and (b) have someone available who could talk about that development. The unit of analysis was the organisation, however three organisations each reported on two programs (with respondents from different sectors). Eligibility was assessed during the phone or email contact (Figure 1). The response rate was 83% (44/53) of eligible organisations, with 47 people interviewed in total.

### Settings and participants

The forty-seven interviews were conducted from September 2012 to May 2013. Those interviewed were located in urban (40%, n = 19), regional (43%, n = 20) and remote (17%, n = 8) localities, with 4–10 organisations in each in each State and Territory in Australia, but with none from Tasmania.

The participating organisations were classified into four groups labelled as follows:

•Twenty-one Aboriginal Medical Services (including one other Aboriginal organisation)– ‘AMSs’ (NB. Services may or may not be ‘community controlled’ so the term is not used here)

•Eight Public Hospitals or Area Health Services and four Government Departments – ‘GOs’

•Six non-government organisations and one Division of General Practice/Medicare Local – ‘NGOs’

•Four universities – ‘Unis’

### Survey instrument

**Figure 2:**
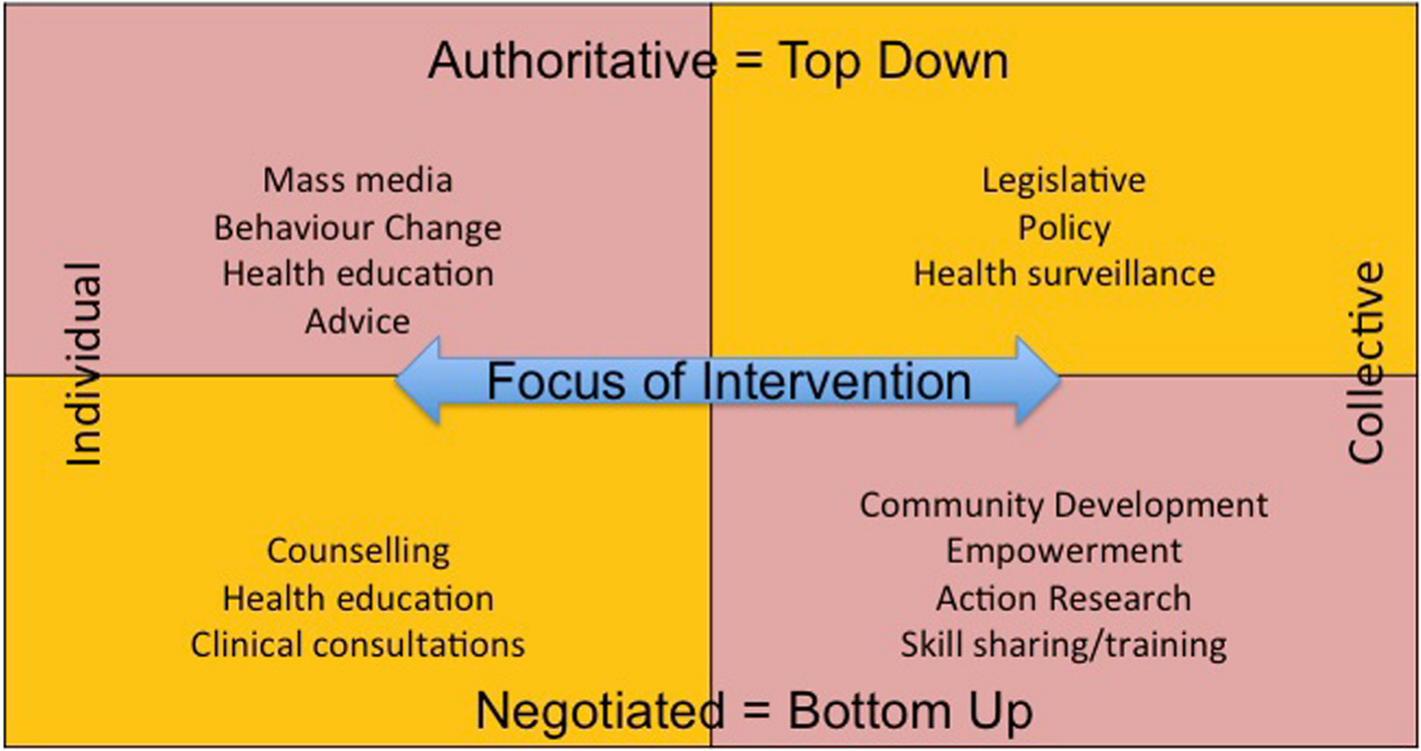
**Beattie’s health promotion model.** (Adapted from Beattie A. [19]).

The questionnaire was based on several frameworks. Beattie’s Health Promotion Model [19], a cross-classification taken from social theory, was used to define whether the messages were created from a top-down approach or bottom-up approach, and if the focus was individual or collective (Figure 2).

To explore the contribution of cultural targeting to message formation, theories from Kreuter et al. and Resnicow et al. were used. Kreuter et al. proposed five strategies [20] to enhance cultural appropriateness (Table 1). Resnicow et al. recommended surface and deep structures for culturally sensitive interventions [21]. Both models were included to analyse how messages were culturally appropriate, and suitably targeted. Table 1 illustrates correspondences between Resnicow’s and Kreuter’s models, and provides definitions.

**Table 1:** Correspondences between theories for tailoring health messages

**Resnicow (2000)**	**Kreuter (2003)**
Surface structure	Peripheral strategies
	Linguistic strategies
	Evidential strategies
Deep structure	Constituent-involving strategies
	Sociocultural strategies

Definitions of terms:

Surface structure: matching materials and messages to ‘superficial’ characteristics of target population.

Deep structure: incorporating cultural, social and historical, mental and psychological forces that influence the targeted health behaviour.

Peripheral strategies: give the appearance of cultural appropriateness by colours, images etc.

Linguistic strategies: make materials more accessible through the use of appropriate language.

Evidential strategies: use epidemiological evidence specific to a population.

Constituent-involving strategies: draw directly on experiences of members of the target group.

Sociocultural strategies: places health issues in context of social and cultural values.

### Survey topics and variables

Table 2 describes variables from the survey, how they were coded or recoded, and which items are reported here. The survey can be obtained from the authors upon request. Most variables were categorical except where indicated. When cells counts were too small for analyses, organisations were classified according to their orientation for Aboriginal and Torres Strait Islander populations or the general population; and/or AMSs versus ‘other’ organisations.

**Table 2:** Variables covered in the questionnaire

**Demographic information about participants and their organisation:**	
•	Location of organisation – coded into urban (RA1), regional (RA2-3), or remote (RA4-5), using ASGC-RA*
•	Role of person in organisation (6 response options e.g. AHW, administrative, researcher)*
•	Organisation type (AMS, hospital/health service, University, research organisation, NGO, GO, other – recoded into AMS, GO, NGO, University)
•	Orientation to general population or Aboriginal and Torres Strait Islander peoples
**General information:**	
•	Overview of program – open ended
•	Adapted or newly made messages (Y/N)
•	Messages as stand alone or part of a program (Y/N)*
•	Target groups (youth, pregnant, elders, adult men, adult women, other)
•	Different message styles for target groups (Y/N)
•	Degree messages developed by a bottom-up vs. top-down approach - scale 1 (mostly bottom-up) to 10 (mostly top-down)
•	Degree messages aimed at individuals vs. community - scale 1 (mostly individual) to 10 (mostly community)
•	Theoretical framework (Y/N – describe if Y)
•	Type of messages (7 response options e.g. educational, threat, positive benefit)
**Formative phases:**	
•	Community consultation (Y/N)
•	How information from the community was gathered (10 response options e.g. community groups, surveys)*
•	Topics explored with community (13 response options e.g. knowledge, threat from smoking, barriers to quit)*
**Message development phase:**	
•	Cultural challenges (Y/N - describe if Y)*
•	Input sources for development (7 response options e.g. community, survey results, expert advice)*
•	Personnel used for advice (10 response options e.g. AHWs, other health professionals, health promotion advisors, Indigenous artists) – recoded into Indigenous advisors (Y/N)
•	Message features (16 response options e.g. Indigenous theme, health related statistics, effect of tobacco on family) - recoded into number of superficial and deep structures (see text)
•	Recommended actions (Y/N)
•	Recommended actions if Y (7 response options e.g. quit smoking, see GP, ring Quitline) recoded into referral options <2 or ≥2
**Pre-test phase:**	
•	Pre-tests with community (Y/N)
•	How pretested (8 response options e.g. informal discussion, reference group, survey)
•	Unexpected outcomes (Y/N - describe if Y)*
**Resource development/distribution:**	
•	Developed resources (Y/N)*
•	Community consulted about resources (Y/N)*
•	Resources developed (15 response options e.g. posters, DVD, T-shirts) recoded into print media, digital media, TV ads, merchandise, resources for quit groups, training, and other*
•	Area of distribution (5 response options e.g. local, regional)*
**Evaluation of messages/resources:**	
•	Messages/resources tested or evaluated (Y/N). ‘Evaluations planned’ were formulated from notes of discussion about evaluation when N was indicated
•	What tested (8 response options e.g. knowledge, quit rates, smoke-free spaces)*

Legend: AMS = Aboriginal Medical Service; GO = government organisation; NGO = non-government organisation; Y = yes; N = no; RA = remoteness area classification; AHW – Aboriginal Health Worker. ASGC-RA = Australian Standard Geographical Classifications - Remoteness Area [22]. *Indicates findings not reported here – for further information contact author or refer to full report [23].

Participants were asked about the incorporation of 16 possible features into message development (responses were yes/no). These features were recoded as either surface or deep structure [21] for analysis. Surface structure included access (e.g. legible print, font, reading age), local languages, use of slang, the ‘look’ or design of the message, and the use of demographic data perceived by the respondents to be pertinent to the target group. Deep structure included Indigenous cultural beliefs, holistic wellbeing, family messages, story-telling, Indigenous role models and community Elders. The number of surface features (out of a possible 11) and the number of deep features (out of a possible 5) reported by the organisations were recorded.

### Community consultation

A community consultation was held with six Aboriginal health staff, and one non-Indigenous manager at two health services, in Queensland and NSW. The consultation process tested face and content validity, acceptability and feasibility of the survey instrument from an Indigenous perspective. The questionnaire was then refined and amendments approved by the ethics committee.

### Procedure

The questionnaire was administered by telephone by the first author. The responses and notes were entered into a secure survey website. Informed consent was obtained, and issues of confidentiality and anonymity were discussed. James Cook University Human Research Ethics Committee provided approval (reference H4466).

### Analysis

Data were analysed with SPSS version 20. Categorical variables were analysed using Pearson chi-squared tests; Fisher’s Exact tests were used when expected cell counts were less than five. For example, we tested the association between organisation type (categories: AMSs and ‘others’ combined) and advice to quit (Yes or No). Kruskall Wallis tests were used to determine association between organisation types (AMS, GO, NGOs and Unis) and the number of deep structures used. Differences between organisational types and the frequency of deep structures used were assessed by pair-wise comparisons (Mann Whitney U tests), and the Bonferroni-Holm correction test for multiple comparisons. The correlation between frequency of deep structures and the reported ratings of Beattie’s axes (Bottom-up to Top-down) was analysed with the Spearman rank correlation coefficient. Non-linear categorical principal component analysis (with the CATPCA program) was also conducted [24] in order to reveal the most efficient and meaningful classifications and relationships among variables and organisations.

## Results

### Program overview

The anti-tobacco messages were reported as being mostly developed for mass media or social marketing i.e. TV, radio, and other media, and/or part of a program. Programs included individual or group cessation, education, and health worker training. Some organisations (19%, n = 9) had adapted other programs to their local community.

### Descriptive and non-parametric analysis

Table 3 shows the message characteristics of the four organisations types. AMSs were more likely to target youth (X^2^ = 9.10, df = 1, p < 0.005) and advised quitting in their messages (X^2^ = 5.16, df = 1, p < 0.05) than the other organisations when their data were combined.

**Table 3:** Message characteristics of organisation types

***Variable***	***Total***	***AMS***	***GO***	***NGO***	***Uni***
	***N = 47***	***N = 22***	***N = 13***	***N = 8***	***N =4***
	***n (%)***	***n (%)***	***n (%)***	***n (%)***	***n (%)***
**Target**					
*Youth target*	30 (64%)	19 (86%)	6 (46%)	3 (38%)	2 (50%)
*Pregnancy target*	26 (55%)	11(50%)	9 (69%)	4 (50%)	2 (50%)
*Adults target*	24 (51%)	12 (55%)	8 (62%)	4 (50%)	1 (25%)
*Elders target*	15 (32%)	10 (45%)	3 (23%)	1 (13%)	1 (25%)
**Theory used**	26 (55%)	9 (41%)	10 (77%)	5 (63%)	2 (50%)
**Community consultation**	45 (96%)	21 (95%)	12 (92%)	8 (100%)	4 (100%)
*Indigenous advisors*	46 (98%)	22 (100%)	12 (92%)	8 (100%)	4 (100%)
**Message Type**					
*Educational*	35 (75%)	16 (73%)	7 (54%)	8 (100%)	4 (100%)
*Positive Benefit*	41 (87%)	21 (95%)	10 (77%)	6 (75%)	4 (100%)
*Threat*	18 (38%)	9 (41%)	3 (23%)	4 (50%)	2 (50%)
*Advocacy*	37 (79%)	19 (86%)	8 (62%)	7 (88%)	3 (75%)
**Action recommended**	44(94%)	22 (100%)	11 (85%)	7 (88%)	4 (100%)
*Referral options ≥2*	31 (66%)	16 (73%)	7 (54%)	5 (63%)	3 (75%)
*Recommend to Quit*	33 (70%)	19 (86%)	7 (54%)	3 (38%)	4 (100%)
**Pre-tested**	34 (72%)	18 (82%)	7 (54%)	5 (63%)	4 (100%)
**Evaluation**	25 (53%)	7 (32%)	7 (54%)	7 (88%)	4 (100%)

Legend: AMS = Aboriginal Medical Service; GO = government organisation; NGO = non-government organisation; Uni = university.

Organisation type and deep structure for message tailoring were significantly associated (p < 0.05). The quantity of deep structures used by AMSs was significantly greater than GOs (p < 0.05) and NGOs (p < 0.05). Organisations that reported using a bottom-up approach (see below about Beattie’s model) were also significantly more likely to use deep structures for messages (r = 0.463, p < 0.001).

Organisations other than AMSs (combined) were significantly more likely to report evaluating programs than AMSs (X^2^ = 7.59, df = 1, p < 0.01). Similarly, when organisations were divided according to orientation to Aboriginal and Torres Strait Islander populations or the general population, the latter were significantly more likely to report evaluating their programs than organisations oriented to Aboriginal and Torres Strait Islander populations (X^2^ = 13.6, df = 1, p < 0.0005). However there were moderating structural issues. Many AMSs stated they were in the early stages and had not yet evaluated their programs. Taking this into account, 72% (n = 34) of organisations reported ‘evaluated or planned evaluations’ , and no significant differences then remained between AMSs and the ‘other’ organisations combined. However when organisations were divided according to orientation to Aboriginal and Torres Strait Islander populations or the general population, the association remained significant (X^2^ = 7.13, df = 1, p < 0.05).

### Beattie’s health promotion model analysis

**Figure 3:**
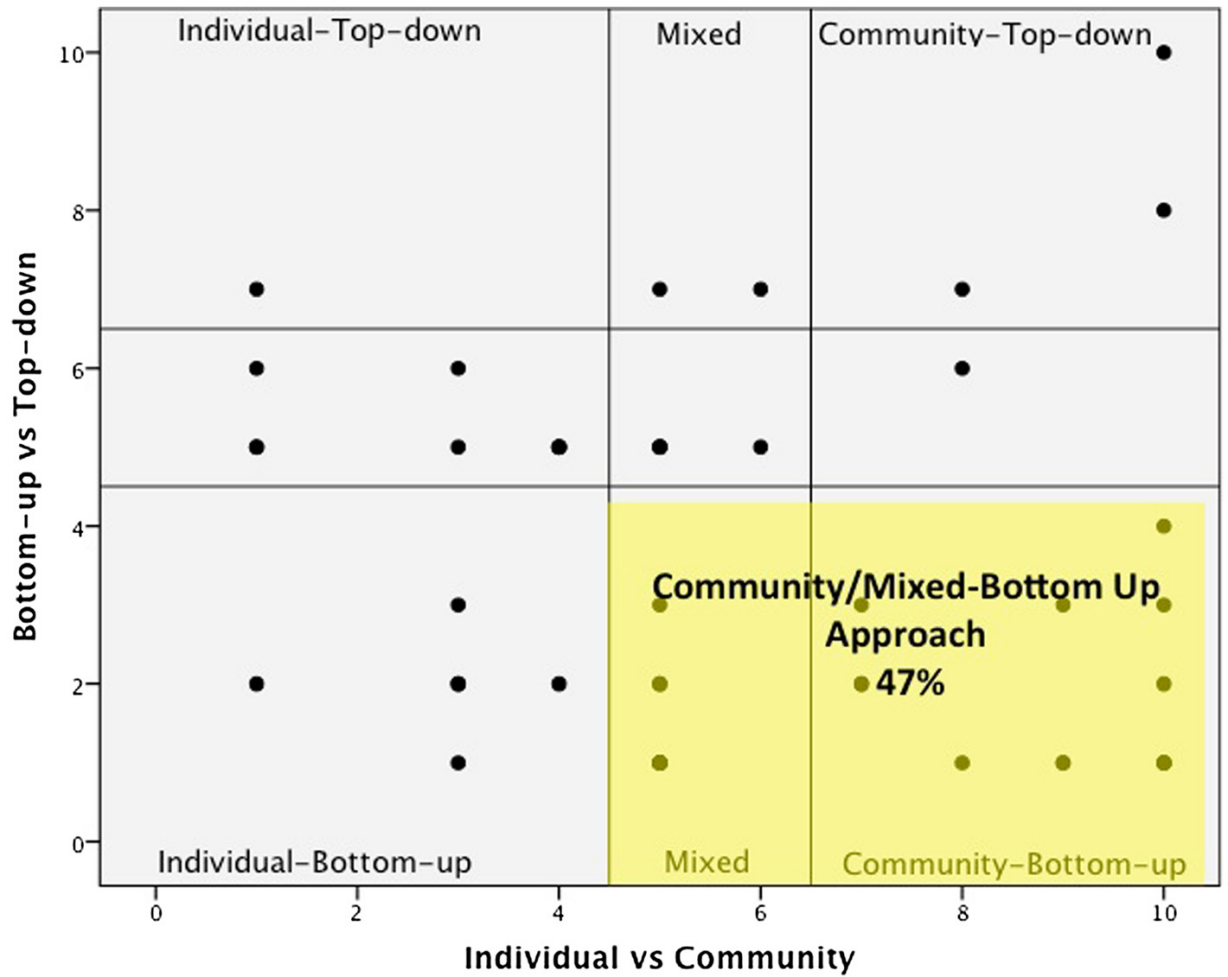
Scatter-plot of ratings of bottom-up vs. top-down and individual vs. community approaches.

Figure 3 shows a plot of the ratings given by the participants when they estimated the degree to which their organisation had used a bottom-up versus top-down approach, and an individual versus community focus. The y-axis of Figure 3 denotes the continuum between a top-down (authoritative) and bottom-up (negotiated) approach. The x-axis represents a spectrum of an individual versus a collective focus for messages. Quadrants were divided up as per Beattie’s Health Promotion Model, but allowed for central sectors when participants estimated ratings of 5 or 6 on either dimension (designated here as a ‘mixed approach’). Sixty percent (n = 28) of organisations reported using a bottom-up approach; 68% (n = 32) of organisations used either a mixed or community approach. When the dimensions were cross-tabulated, the Community-Bottom-up category (includes ratings in the central sector for mixed community/individual focus) was the largest single grouping reported (47%, n = 24). Community-Bottom-up approaches are commonly termed empowerment or community/participatory development models [19].

### Categorical Principal Component Analysis (CATPCA)

To examine potential associations between categorical and numerical variables, we used CATPCA. In a first iteration, initial inclusions were organisation type, orientation, rurality, theory used, individual vs. community approach, top-down vs. bottom-up approach, message features, deep and surface message structure, recommended actions, referral options, pretests, evaluation, evaluations done/planned, unexpected outcomes, and cultural challenges. When eight of the most relevant items were retained for analysis, based on factor loading scores of >0.4, the CATPCA revealed a parsimonious two-dimensional model with eigenvalues of 2.34 (dimension 1) and 1.79 (dimension 2), together accounting for 53% of the variance in the data.

**Figure 4:**
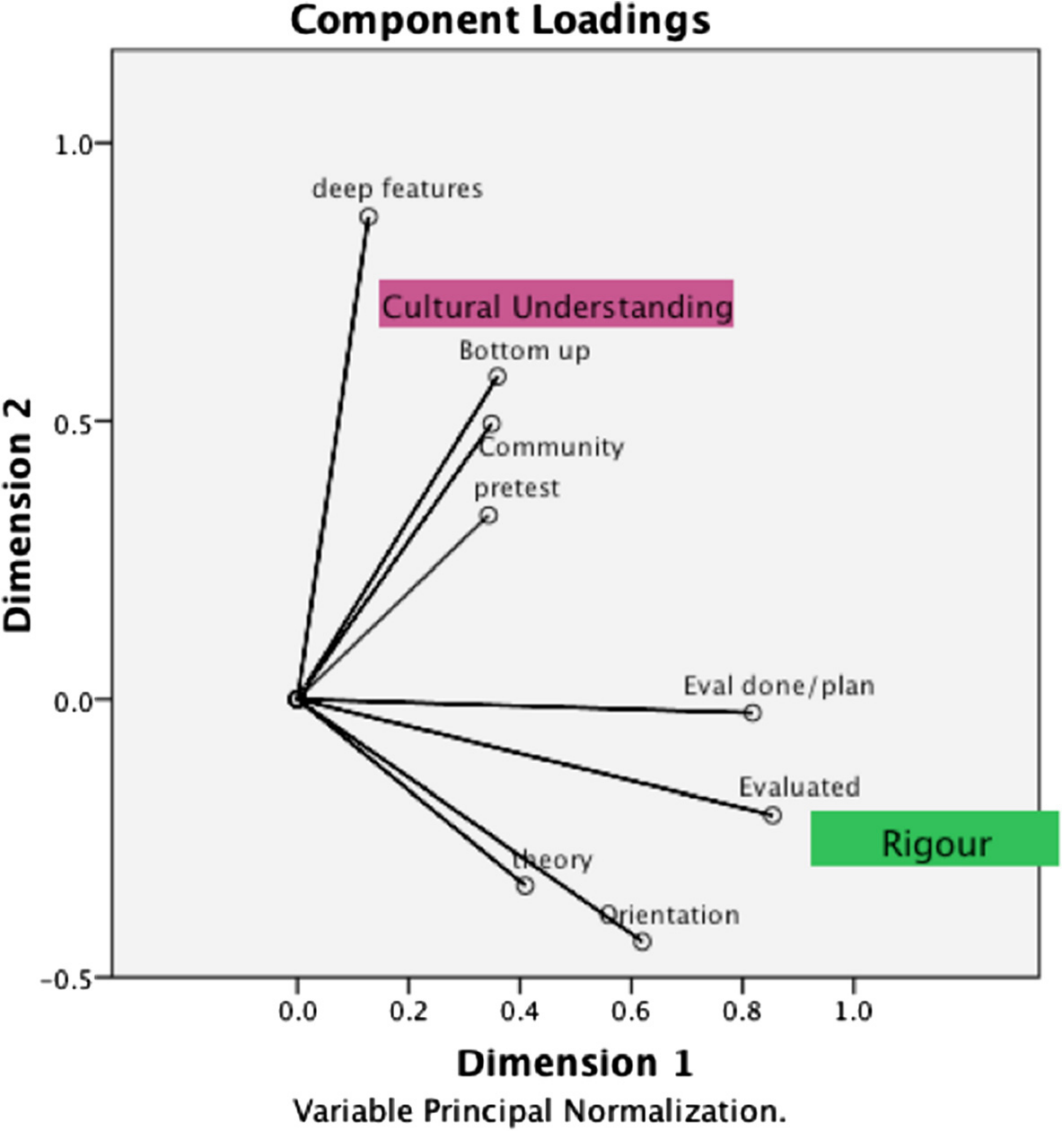
**Component loadings from CATPCA on eight retained variables.** Component loadings as Pearson correlations range between -1 and 1 in this two dimensional solution. The variables form two groups defined here as “Cultural Understanding” and “Rigour”. The co-ordinates of the end point of each vector are given by the loadings of each variable on the first and second dimensions. The variables closely grouped together in the plot are positively related. Vectors making a 90-degree angle indicate they are not related. Legend: ‘Community’ = Individual vs. community orientation; ‘Bottom-up’ = Bottom-up vs. Top-down approach; ‘deep features” = numbers of deep message features; ‘pretest’ = a pretest was conducted; ‘eval done/planned’ = an evaluation was either completed or planned; ‘evaluated’ = evaluation was completed; ‘theory’ = a theoretical framework was used; ‘orientation’ = the organisation usually served the general population vs. the Aboriginal and/or Torres Strait Islander populations.

Figure 4 shows the plotted coordinates for the retained variables, and how the variables relate to one another and to the two dimensions. We named these two principle categories “cultural understanding” and “rigour”. The variables ‘Community’ , ‘Bottom-up’ , ‘deep features’ and ‘pretest’ are grouped high on dimension 2 and in the lower range of dimension 1. (The ‘surface features’ variable was also in this group, but was removed as it obscured the other vectors). The other items coded as ‘evaluated’ , ‘eval done/plan’ , ‘theory’ , and ‘orientation’ are grouped on the positive scale of dimension 1 and low on dimension 2. The items ‘pretest’ and ‘theory’ are closer to the centroid (0), which means they contributed less to the overall variance.

**Figure 5:**
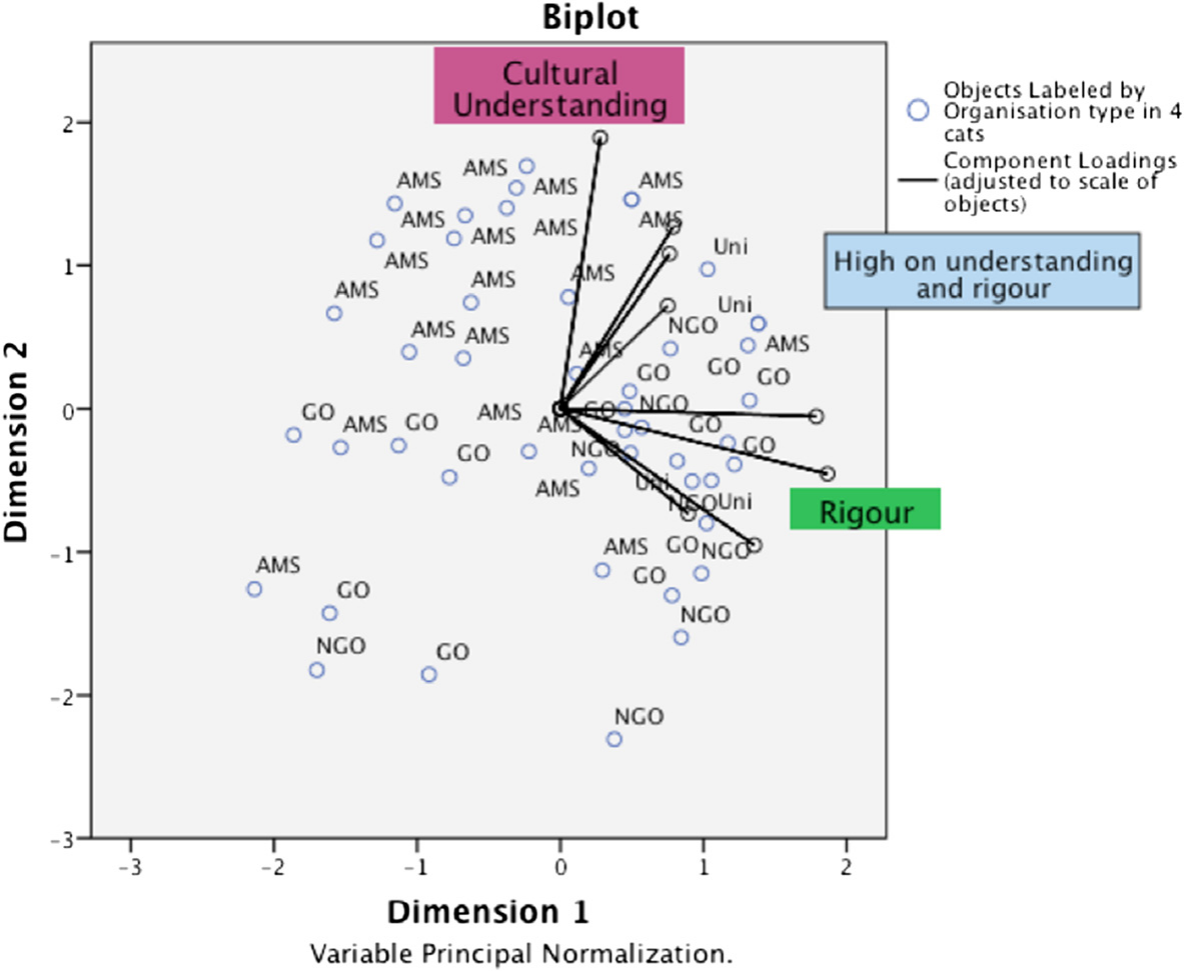
**Biplot of the organisation types.** Objects represented by circles and the component loadings indicated by vectors from the CATPCA solution on the eight variables. The vectors for the component loadings have been adjusted to the range of the objects for clarity. The organisations that are plotted between the two ‘arms’ of the vector groups are high on both “cultural understanding” and “rigour”. (Variables omitted). Legend: cats = categories; AMS = Aboriginal Medical Service; GO = government organisation; NGO = non-government organisation; Uni = University.

Figure 5 shows a scatter plot with each organisation (labelled by organisation type) plotted against the two dimensions. A large cluster of AMSs scores high on dimension 2 (cultural understanding). Some ‘other’ organisations (GOs, NGOs, and Unis) are located high on dimension 1 (rigour) but low on dimension 2 (cultural understanding). Organisations that are high on both dimensions (between the two principal component axes in the right upper quadrant) combine both cultural understanding and rigour, and may be exemplars for the development of anti-tobacco messages. These include a range of organisations of all types. A few outliers, in the bottom left quadrant of the figure, are low on both components.

## Discussion

Our unique study examined the contemporary factors reported to contribute to the development of anti-tobacco messages for Aboriginal and Torres Strait Islander communities. The survey revealed differences in how organisations were developing messages for the target populations. The CATPCA revealed a parsimonious model with two components interpreted as “cultural understanding” and “rigour” explaining the majority of variation in findings. AMSs were demonstrated as strong in providing cultural understanding through culturally sensitive messages and empowerment approaches. Organisations oriented to the general population demonstrated strength in the use of theory and evaluation. Some organisations of all types provided both aspects, proposed to be best practice.

In regard to the evidence about mass-media tobacco campaigns, there are some cautious comparisons that can be made between Aboriginal and Torres Strait Islander groups and other populations, including those of low socio-economic status (SES) [25]. There is consistent evidence to support the relationship between smoking status and social gradient for Aboriginal and Torres Strait Islander peoples [26], and 57% of Aboriginal and Torres Strait Islander peoples in the lowest three deciles of disadvantage [27]. We outline the literature below about the use of cultural-appropriate messages, the use of theoretical frameworks, recommending people quit smoking, and providing two or more referral options. The findings suggest that some campaigns for Aboriginal and Torres Strait Islander peoples are using these promising features for the development of Indigenous tobacco control programs in Australia.

### Elements for cultural understanding and developing anti-tobacco messages

Community consultation was engaged in by nearly all the organisations, irrespective of approach. Most organisations used bottom-up approaches (60%), which are more likely to be empowerment models [28]. Empowerment approaches are time-consuming and may not be achievable within limited project frameworks [28]. The focus on combined individual and collective interventions is supported by other researchers [29,30], with 68% in our study using a community or mixed focus. Customisation of health messages applied at the community level, have the potential for wider reach [29]. A recent systemic review suggests that low SES groups require higher exposure to anti-tobacco messages to effect the same changes as mid-high SES groups [5], so campaign reach is critical.

Both surface and deep structures are important to facilitate the recipient’s experiences of self-referencing and identification with messages [31]. Surface structures relate to the ‘fit’ of the message [21]. Surface structures, by being peripherally processed, are useful in those less motivated, however deep structures, more centrally processed, produce longer lasting effects [29]. The finding that the AMSs used more of the deep structures for cultural sensitivity (such as Indigenous world view, spiritual, cultural and family values) relates to message ‘salience’.

Media messages also encourage quitting through social networks [5]. The use of social networks is relevant to the local approaches taken in Aboriginal and Torres Strait Islander communities. ‘Real stories’ , used here by several organisations, encourage dialogue amongst smokers and have been effective in low SES communities when paired with information on where to seek help [32].

There is inconsistent evidence about whether threat-based messages are suitable to motivate Aboriginal and Torres Strait Islander peoples to quit. Qualitative research has revealed some avoidance of fear-based messages by Aboriginal people [33,34]. Conversely strong graphic images and those featuring an ill person, have been rated highly by Aboriginal and Torres Strait Islander smokers under experimental conditions [8]. Organisations here used fewer threat-based messages than positive appeals or combined approaches. No organisations used only a threat approach. Threat messages in isolation are less effective for the general population than when combined with a positive approach [29]. For disadvantaged and low SES smokers, negative health effect themes (including testimonials and graphic depictions) are effective [5], especially when combined with how-to-quit messages, but how-to-quit messages on their own are less successful [5]. Recommending two or more referral options, reported here by 66%, has been shown to positively influence message efficacy [29].

There is evidence that targeting youth through media and multi-component community interventions are both effective approaches to prevent smoking uptake [35,36]. As yet there is insufficient research available to be certain these approaches prevent initiation by Indigenous youth [37]. The prominent approach towards youth revealed in our study is pragmatic as young people represent such a large percentage of Aboriginal and Torres Strait Islander communities.

Many organisations in this study used media messages within the context of comprehensive health promotion approaches fostering empowerment: approaches successful in other populations [5,38]. Media campaigns appear to be most effective among low SES smokers when implemented alongside comprehensive programs that include community mobilisation, free access to nicotine replacement therapy, social support and policy changes to transform the social context of tobacco use [25]. Low SES smokers may have less opportunity to support long term abstinence, compounded by low access to services [25]. It is not yet determined whether comprehensive programs are going to be effective for Aboriginal and Torres Strait Islander peoples. Issues of intervention fidelity also need to be taken into account.

### Elements for rigour in developing anti-tobacco messages

A good evidence base or assessment of program theory is recommended to avoid an ad hoc process in the development of health promotion approaches [38]. The use of a theoretical basis for message development by more than half of the organisations is promising, as programs that use a higher number of theoretical concepts for tailoring have shown larger effect sizes [29]. Cultural tailoring also improves the impact of theoretical tailoring [39]. Most organisations we surveyed promoted behaviour changes, and many took local and cultural demographical information into account. When these approaches are combined they can produce cumulative effects [29].

Evaluation is important to determine whether objectives have been realised [38], and to build an evidence-base for future interventions, and research translation. Rigorous evaluations are needed to build the evidence base around current tobacco action initiatives in Aboriginal and Torres Strait Islander communities. Carson et al. point to the need for methodological rigour in research that runs alongside community tobacco programs, with adequate control groups, pre and post measures, and meaningful follow-up periods [40]. Resources for anti-tobacco campaign evaluation are available [41]. Evaluations to consider include formalised pre-tests, process evaluation, campaign awareness and recall, community involvement and reach, changes in knowledge, attitudes and beliefs, behaviour change (with standardised and/or validated quit rates [42]), smoke-free behaviours, and access to cessation services.

Most public health strategies for Aboriginal and Torres Strait Islander communities however are not evaluated, inadequately funded, not sufficiently robust to measure impacts, and not published [43]. This is a structural imbalance that is essential to address [44]. The unique methodological challenges involved in evaluating Indigenous programs, summarised by Cobb-Clarke [45], include small sample sizes which limit the power to detect slight changes, difficulties with appropriate randomisation, and limited meaningful control groups due to the diversity of Indigenous communities. Some communities may be the recipients of several policy-driven multi-faceted programs, so isolating the individual effects of these are challenging. Organisations therefore need to be very well resourced to evaluate program outcomes with dedicated funding, adequate time frames, human resources and expertise. This may include building research capacity.

### Recommendations

Our recommendations for cultural understanding-rigour, pending further evidence, include the following:

•Organisations should consider both cultural understanding and rigour in their planning to guide development of anti-tobacco messages for this population.

•Deep structure for messages should be considered early in the project plan, as there may be limited opportunities to redress earlier omissions.

•We recommend synergy of action through partnerships. As organisations have different strengths and capacities, partnerships with organisations from different sectors is pragmatic. However community-based bottom-up approaches (empowerment models) should be maintained throughout.

### Strengths of the study

The study, we believe, has effectively captured current strategies occurring Australia-wide. Due to the high response rate, and the contribution of organisations from nearly all States and Territories, spread across urban, rural and remote areas, the study is likely to be transferable to other organisations engaged in Aboriginal and Torres Strait Islander community tobacco control in the near future. CATPCA has enabled us to propose a theoretical model of cultural understanding-rigour as two important components to be considered when planning anti-tobacco messages and programs.

By providing an Australian snapshot of current anti-tobacco message development for Aboriginal and Torres Strait Islander peoples this research contributes to the following priorities of the National Partnership Agreement on Closing the Gap on Indigenous Health [16]and the National Tobacco Strategy tobacco control objectives [17]:

•Addresses populations with high smoking rates

•Furthers research to guide Indigenous tobacco control policies

•Guides culturally targeted message development through social marketing, mass media and smoking cessation interventions

•Highlights the importance of evaluating tobacco control programs alongside the roll-out of state, territory and national programs

•Recommends strengthening collaboration between Aboriginal and Torres Strait Islander and other organisations

### Limitations

Selection bias is likely, as some organisations could not be assessed for eligibility. Organisations may have been missed if their programs were not publicised. Data collection relied upon self-report. Although less likely to occur with an interview where probing was possible, inter-subjectivity inevitably may bias information. Outcomes of programs (i.e. effectiveness) were not evaluated. The study could have been improved by recording length of time since inception of the programs.

## Conclusion

The findings demonstrated that organisations in Australia are engaged in developing cultural understanding, fostering empowerment, and responding to the local needs of Aboriginal and Torres Strait Islander communities. Features associated with successful campaigns elsewhere are starting to be used for Aboriginal and Torres Strait Islander peoples. These include the cultural adaption of messages, the use of theory to inform message development, recommending people quit smoking; providing two or more referral options in the messages.

This study has provided new insight into the current development of anti-tobacco messages in Australia. Based on current and recent practices in Australia, we propose a theoretical ‘cultural understanding-rigour’ model as important twin aspects to develop evidence for appropriate tobacco control programs for Aboriginal and Torres Strait Islander peoples. The model of cultural understanding–rigour is yet to be tested by project outcomes, and needs further validation.

Consistent with policy recommendations in Australia [17], persuasive anti-tobacco messages should continue to be used to inform and motivate, as part of comprehensive programs that provide support and services for those attempting to quit. Refinement of evaluation and synergy of action between organisations from different sectors may hasten the goal of closing the gap on Indigenous health caused by tobacco smoking. The future production of guidelines for the development of Indigenous anti-tobacco health promotion programs may facilitate these processes.

## Competing interests

AM receives a personal income from Cancer Research UK via University College London. He has received travel funding, honorariums and consultancy payments from manufacturers of smoking cessation products (Pfizer Ltd, Novartis UK and GSK Consumer Healthcare Ltd). He also receives payment for providing training to smoking cessation specialists; receives royalties from books on smoking cessation and has a share in a patent of a nicotine delivery device.

## Authors’ contributions

GG conceived and designed the study and developed the questionnaire, contributed to the Indigenous community consultation process, conducted the interviews, performed the statistical analysis, and the qualitative analysis, and wrote the manuscript. KW contributed to the statistical analysis, checked the accuracy of the statistical findings, and critically reviewed the manuscript. LS contributed to the Indigenous community consultation process, the questionnaire design and the qualitative analysis, and critically reviewed the manuscript. AM contributed to the study design and questionnaire development, and critically reviewed the manuscript. YC contributed to the interpretation of Indigenous cultural and policy issues relevant to the study. AC contributed to the study design and questionnaire development, recruitment strategy, analysis of data, and interpretation of findings, and critically reviewed the manuscript. All authors read and approved the final manuscript.

## Authors’ information

GG is a non-Indigenous General Practitioner and Tobacco Treatment Specialist involved with developing local Indigenous smoking cessation programs in NSW, Australia. KW is a non-Indigenous senior academic and epidemiologist. LS is a non-Indigenous health promotion researcher in Indigenous health. AM is a non-Indigenous expert and researcher in tobacco treatment and services. YC is an Aboriginal elder and senior academic. AC is a non-Indigenous senior researcher of community-based Indigenous substance use and tobacco programs.

## Pre-publication history

The pre-publication history for this paper can be accessed here:

http://www.biomedcentral.com/1471-2458/14/250/prepub
